# Using Grounded Theory to Identify Online Public Opinion in China to Improve Risk Management—The Case of COVID-19

**DOI:** 10.3390/ijerph192214754

**Published:** 2022-11-10

**Authors:** Chao Zhang, Ning Ma, Guohui Sun

**Affiliations:** 1 Publicity Department of Party Committee, Beijing University of Technology, Beijing 100124, China; 2 Graduate School, Communication University of China, Beijing 100024, China; 3 Beijing Key Laboratory of Environment and Viral Oncology, Faculty of Environment and Life, Beijing University of Technology, Beijing 100124, China

**Keywords:** risk management, COVID-19, online public opinion, epidemic prevention and control measures, real public opinion

## Abstract

Background: During the outbreak of COVID-19, online public opinion related to the epidemic was rapidly generated and developed rapidly. If some online public opinions cannot be effectively responded to and guided, it will bring risks to social order. The government should understand how to use information on social media to grasp public demands, provide useful information in a timely manner and take countermeasures. Studying the formation mechanism of online public opinion during the outbreak can help the government make scientific decisions and improve risk management capabilities. Methods: The research selects the public opinion information of online platforms represented by WeChat, online communities, Sina Weibo and search engines, involving 75 relevant texts (1 January to 31 March 2022). According to the grounded theory method, using the QSR NVivo12 qualitative research software, the collected network texts were successively researched using open coding, axial coding and theoretical coding. Results: The structure of online public opinion during the COVID-19 epidemic was obtained. The operation mechanism of the online public opinion system about COVID-19 was mainly affected by the interaction of online public opinion objects, online public opinion subjects, online public opinion intermediaries and government forces. It was based on social facts and citizens’ appeals as the starting point, subject behaviors and prevention and control measures as the focus, government’s governance as macro-control and citizens’ evaluation as the guide. Conclusions: Scientific analysis of online public opinion is an important tool to identify and manage risks and improve the quality of government activities. Online public opinion has the function of assisting government decision-making, and the government can identify the important information reflected in it, especially the mainstream public opinion, as a reference for decision-making. By taking effective measures and properly responding to citizens’ reasonable demands, the government can prevent social risks and avoid new negative public opinions. Contributions: According to the characteristics of the basic model of online public opinion, this study provides risk mitigation suggestions for Chinese public sectors to use online public opinion, optimize epidemic prevention policies and formulate strategic measures.

## 1. Introduction

It has been more than two years since the outbreak of COVID-19 in 2019. At present, the epidemic prevention and control has entered a new stage—the normalization stage [1]. During this period, governments need to adapt to outbreak-related emergencies and find new solutions to unresolved and emerging problems. Identifying online public opinion is one of the key actions of improving risk management from the perspective of optimizing health policy for the best results.

According to the China Internet Network Information Center (CNNIC), as of June 2022, the number of internet users in China was 1.051 billion, and the internet penetration rate was 74.4%, of which 99.6% use mobile phones to access the internet [2]. With such a large number of Internet users, everyone can express their opinions online, and hot events can quickly form a tsunami of online public opinion, especially during the COVID-19 epidemic, which shows that the opinions of internet users have become an important factor for the government to consider. At present, the epidemic situation in China is still facing sporadic outbreaks, which has repeatedly troubled the government and people. Internet users also express their opinions through the internet, some of which are rational and some irrational. If these online public opinions are not effectively identified, emphasized and guided, they will easily cause new social problems, and even lead to social risks [3].

Faced with the current situation in China, this study attempts to answer the following questions. What important factors can be identified in the process of generating online public opinion related to the COVID-19? How do we effectively avoid these risks from online public opinion?

In recent years, various online social media platforms have been widely used, and people have released a large amount of information to the internet, forming a huge online public opinion database over time. During the epidemic, these data covered all aspects of social life, involving vaccination, isolation measures, food security, urban management, medical assistance and other issues, covering social hotspots and difficult issues in a certain period of time, just like a “Microcosm” of social reality [4].Therefore, based on China’s social situation, this study selects representative events that have had an important impact in China during the COVID-19 epidemic, deeply analyzes the constituent factors in the process of generation and change of public opinion, builds a structural model of online public opinion and puts forward relevant suggestions for risk mitigation.

## 2. Literature Review

### 2.1. Research on the Factors Affecting Online Public Opinion

In recent years, some scholars have studied the factors that affect the generation and change of online public opinion [5]. Researchers generally believe that the generation and change of major online public opinion is the product of the comprehensive effect of various social factors. The causes can be explored from the aspects of the subject, object, media, social environment, contingency factors and the manifestation of public opinion information. For example, some scholars believe that the causes of online public opinion include four aspects: political and economic reasons, social environment and hot events, false and bad information dissemination, strong media influence in the new media era, etc. [4,6]. Some scholars also found that opinion leaders, netizens, media and government involvement can affect the attention of public opinion events [7]. In particular, opinion leaders can have an important impact on online public opinion. The government should pay more attention to the scale of public opinion leaders when monitoring and warning, or responding to public opinion in group events. The corresponding government agencies can interact with the news media, form a group, act as the opinion leader, effectively attract public attention and avoid causing new public topics or social risks [8].

Some scholars believe that the influencing factors can be divided into objective factors and subjective factors [9]. Among them, objective factors refer to social and economic factors that do not change with the development of events. For example, unexpected public events will cause a huge psychological impact on the public. Subjective factors refer to the participants of public opinion events, which affect the trend of public opinion due to their different performances. The objective factors mainly include three aspects. First is the problem: internet users pay different attention to different social events. Obviously, topics involving official corruption, social justice, environmental pollution and religious affairs are highly sensitive and can easily influence public opinion. Second, regionality: China has a wide geographical distribution, with unbalanced regional population distribution and economic development. The connectivity, timeliness and wide use of internet technology enable everyone in any region to access, disseminate and edit information equally. However, in the economically developed and densely populated eastern region, people are more inclined to participate in the discussion of network events. Third, the impact of the original event: if the original event attracted the attention of many netizens, media reports, and did not receive a timely response from the government, it may escalate into a large-scale public opinion event [10].

### 2.2. Research on the Changing Law of Online Public Opinion

Some scholars believe that the change of online public opinion has a periodic law, and each stage presents different characteristics [11]. It is pointed out that online public opinion mainly has three stages: the occurrence stage, propagation stage and decline stage. In each stage of public opinion propagation, the government can introduce judicial or government official nodes with high authority and credibility in the center of the public opinion propagation network, strengthen the interaction between the government and the public, and establish effective communication channels with internet users. At this time, relevant institutions can monitor the occurrence of relevant events and try to avoid the occurrence of secondary negative public opinion [11]. There are also different views that the transmission of online public opinion has four stages: focus, formation, climax and resolution [12]. The view that there are four stages in the transmission of online public opinion is that there will be an incubation period before the outbreak of online public opinion [13].

### 2.3. Research on the Impact of Internet Public Opinion

In recent years, China has been in a period of social transition, with various hidden social contradictions, and the existence of multiple interest groups has the potential to bring about divergent interests and conflicting opinions, which will cause various public incidents and affect state security and social stability [14,15]. The breeding and spreading of negative emotion in public emergencies posed severe challenges to social governance [16]. During the development and evolution of COVID-19, the formation and development of public opinion information has been dynamic and constantly changing under the influence of many factors: some public opinions are positive, but many are destructive and antisocial, difficult to predict and control, and can easily bring social unrest. Based on this situation, as the leading force of public opinion on social networks, the government should pay attention to controlling and guiding public opinion on the Internet.

Researchers mainly focus on the impact of online public opinion on such aspects as public decision-making, political democracy and social stability. In terms of the impact on public policy, there are both positive and negative impacts. Relevant departments can make decisions based on online public opinion on the basis of data collection and information research and judgment to create a favorable environment for public decision-making and implementation. False or disorderly development of online public opinion will affect the public decision-making and implementation of the government [17,18,19]. In terms of the impact on democratic politics, the network, as the most active and simple and direct public opinion expression channel, can change or affect the traditional political power structure and discourse expression mode. For example, it is the trend of online public opinion supervision function to change from one-way supervision to two-way interaction with institutionalized anti-corruption [20,21]. Online public opinion also has a certain impact on social governance. Internet public opinion is an important feedback mechanism for the government’s risk management, and it is a “mirror” for the government to understand the people’s expectations and summarize political gains and losses [22]. By analyzing online public opinion data, checking and filling gaps in the process of public opinion screening, and taking targeted improvement measures, it is conducive to promoting the scientific and democratization of government risk management, and promoting governance innovation and improvement [23,24].

### 2.4. Research Review

Through the literature review on the influencing factors and propagation rules of online public opinion, it was found that the research on the influencing factors of new coronavirus online public opinion is mostly concentrated in computer science and other fields. These studies usually analyze online public opinions from the perspective of data modeling [25,26,27]. At present, there is a lack of systematic identification and risk interaction research on risk factors of COVID-19, and the research on risk transmission mechanism of online public opinion of COVID-19 is relatively insufficient. In addition, the research on risk transmission mainly focuses on information management and news communication. There are many studies on the impact of online public opinion, but there are few studies on how to identify and analyze the influencing factors, and then rationally respond to the epidemic risk. Therefore, this study aims to study and identify the influencing factors and development and change laws of online public opinion from the background of epidemic normalization, and provide a reasonable proposal for optimizing epidemic prevention strategies and resolving social risks.

## 3. Research Design

### 3.1. Methods

Grounded theory is inductive in its nature and “facilitates the process of discovery” or “theory generation” [28]. The theory develops through an iterative process of interviewing and the constant comparison and analysis of data. It encompasses a constructivist approach which enables the searcher to consider published literature prior to conducting research as something that can enhance the process, rather than forcing preconceived ideas on the emerging theory. Grounded theory enables the researcher to address the experiences of the participants as well as acknowledge the interpretations by the researcher and it takes full account of the researcher’s position within the process that leads to the emerging theory [29,30].

**Figure 1:**
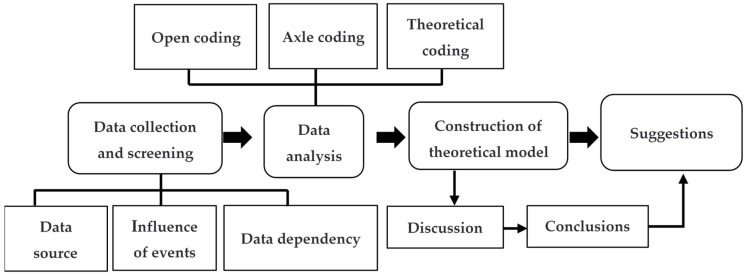
The research flow chart.

According to grounded theory, the collected text was coded and analyzed by using the QSR NVivo12 qualitative research software (https://www.qsrinternational.com/nvivo-qualitative-data-analysis-software/home, accessed on 22 March 2022). This study focuses on important online public opinion events in China under the background of normalization of epidemic prevention and control, uses grounded theory methods to study the key risk factors and change rules in online public opinion events and constructs a theoretical model of online public opinion transmission. Furthermore, the research focuses on primary data, generating concepts from massive raw data. Connections are made by comparing concepts in detail, ultimately building theoretical models based on coding. This paper mainly adopts open coding, axial coding and theoretical coding to complete the qualitative research on the online public opinion text data of COVID-19 (shown in Figure 1). Through text coding analysis, we can distinguish the focus of various types of online public opinion, identify the direction, mode and development trend of public opinion, and reflect the will of citizens. We can also understand the evaluation of epidemic prevention and control policies expressed by online public opinion, and then provide suggestions for optimizing government risk management.

### 3.2. Sample Selection

Some scholars have found that the information reflecting online public opinion comes from the information focused on by major network platforms within a certain period of time [31,32,33]. In mainland China, online public opinion in a specific period of time mainly comes from commentary articles on the WeChat platform, heated discussions on Sina Weibo, posts and comments in online communities and popular search engine information rankings. The evolution of online public opinion related to COVID-19 is relatively complex, while the development of public opinion also presents a certain “life cycle” feature [34]. The information evolution of these major online platforms can basically reflect the various processes of the dynamic evolution of online public opinion [35]. Therefore, the selection of data objects in this study mainly includes four aspects: articles corresponding to WeChat hot words, posts on the online communities post ranking list, event information corresponding to Weibo hot topics and event information corresponding to search engine hot words.

The Public Opinion Data Center (PODC) of People’s Daily Online publishes the top ten pieces of online public opinion information among the four information sources every week.

The PODC releases the ranking information to specific paying users and does not allow users to disclose relevant information. The ranking information is obtained through certain data analysis. The information ranking list of Wechat is a comprehensive ranking based on the search of Wechat hot words. The information ranking list of online communities is a comprehensive ranking based on factors such as the number of hits, replies and social attention of Chinese knowledge communities such as the Zhihu community, Hongwang community and the Huasheng forum. The Weibo information ranking list is a comprehensive ranking based on popular topics on microblogs. The information ranking list of the search engine is a comprehensive ranking of the latest data collected by heat filtering and directional crawling through the public opinion channel of the People’s Daily Online, the monitoring system of Chinese newspapers and periodicals of the People’s Daily Online, Baidu, China Search and Google Advanced Search. The object data come from national and regional important news websites, online communities and blogs.

**Figure 2:**
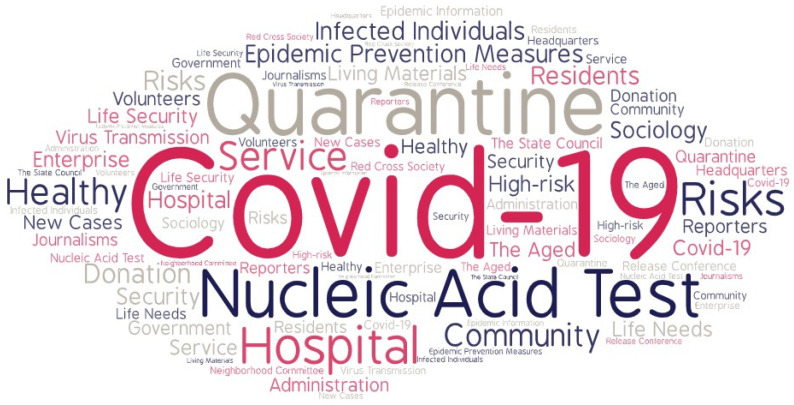
High-frequency words in online public opinion information.

This study selected the information related to the COVID-19 from the four information sources published by PODC from 1 January to 31 March 2022, involving 75 relevant texts, with a total of 74,360 words (using big data analysis, the high-frequency words as shown in Figure 2 and Table 1). For example, as shown in Table 2, the online public opinions related to COVID-19 of the main network platforms from 25 to 31 March 2022 released by PODC were selected. First, the representative WeChat official account tweets about COVID-19 were selected for analysis, such as “children dance for epidemic prevention workers to thank, netizens: it’s better to get home quickly after finishing” (28 March 2022) [36]. Secondly, the representative posts of major online communities, such as “Cap the cost to make COVID-19 antigen testing more affordable” (28 March 2022), were chosen for analysis [37]. Thirdly, the representative Sina Weibo’s reports on COVID-19 were selected for analysis, for example, “a nurse in Shanghai died of asthma: the emergency department of Shanghai Oriental Hospital was temporarily closed due to epidemic disease” (25 March 2022) [38]. Fourthly, we selected the popular search contents related to the COVID-19 from the search engine, such as “Changchun apologizes to the public for the difficulty of buying vegetables: we have organized multi-channel efforts to increase stock” (29 March 2022) [39]. The samples were selected mainly for the following reasons: (1) these online public opinion events are all related to the COVID-19, which have aroused widespread concern and discussion in China, and have a huge impact on society. (2) These cases are highly typical and representative. They are all data collected by PODC through heat filtering and targeted capture, and they are ranked in the top ten of the weekly ranking according to the attention index published by PODC. (3) In terms of data availability, these data are from official media and influential We Media, including academic research results, expert analysis, public comments, party interviews, stakeholder interviews and other key data, which provide sufficient data for this study.

## 4. Results

### 4.1. Open Coding

Open coding is a key link in qualitative research. By coding meaningful expressions in online public opinion texts, a node of related “concepts” can be created. For example, “guaranteeing the price stability, quality safety and market supply of daily necessities, and ensuring market stability during the epidemic prevention and control period” is a node of “stabilizing the market order”. During the encoding process, the effective information of each sentence is captured and the “concepts” with a small number of nodes are excluded. Through coding, a total of 28 meaningful “concepts” and 572 nodes were obtained. Open coding example information is shown in Table 3.

### 4.2. Axle Coding

Axial coding forms higher-level categories through theoretical logic to cover already formed concepts, and constitutes an important link in refining theory from qualitative data. This study focuses on the analysis of online public opinion related to the epidemic, and finds that the 28 first-level concepts generated by text can be further divided into 6 categories. Specifically, as shown in Table 4, concepts such as “changes in the epidemic situation” and “social order status” belong to the objective social situation during the epidemic and can be classified as social facts. Concepts such as “nucleic acid testing requirements” and “medical services demands” are the specific needs of citizens during the epidemic and can be classified as citizens’ appeals. The concepts such as “official’s enforcement actions” and “citizens’ rights protection actions” directly reflect the behavior activities of different subjects during the epidemic period, and can be classified into the category of subject behaviors. “Investigating epidemic information” and “taking prevention and control measures for specific areas” are all specific measures for epidemic prevention and control, which can be classified as prevention and control measures. “Negative evaluation” and “positive evaluation” are comments made by public opinion on a phenomenon or behavior during the epidemic, which can be classified as citizen’s evaluation. The concepts of “formulating prevention and control policies” and “strengthening the support of epidemic prevention and control” are measures taken by the government during the epidemic period, which can be classified as government governance. Therefore, through repeated analysis and research on the concept, a total of 6 secondary categories were obtained, namely, social facts, citizens’ appeals, subject behaviors, prevention and control measures, citizen’s evaluation and government’s governance. These categories generally cover the focus of public opinion during the epidemic, thus can objectively reflect the reality of online public opinion.

### 4.3. Theoretical Coding

Theoretical coding is the process of further generalizing the obtained categories to form a theory through induction and innovation. Through the analysis of six categories, it was found that the number of concepts contained in “citizens’ appeals”, “subject behaviors” and “prevention and control measures” ranked in the top three of the six categories, accounting for 64%, and became the most important part of the online public opinion system. The evolution of the whole online public opinion event is inseparable from the dynamic relationship between these three categories. Citizens’ appeals are an important part of the epidemic prevention and control that can directly reflect the opinions of the majority of netizens. Online social media has become a platform for social management and for netizens to express their opinions and intervene in public affairs. Whether the citizen’s appeals, discovered from online public opinion, can be effectively responded to is related to the effect of social governance. The subject behavior includes the behavior of different organizations and individuals in the context of the epidemic, including legal behavior and illegal behavior. These behaviors have a direct or indirect impact on social order and the effectiveness of epidemic prevention and control in the context of the epidemic. The behavior of different subjects will affect the way the government is governed, as well as the evolution of specific prevention and control measures. Epidemic prevention and control measures are usually specific actions taken by relevant government departments and personnel, and are a dynamic behavioral system. Good prevention and control measures will have a positive effect on citizens’ appeals and subject behavior.

**Figure 3:**
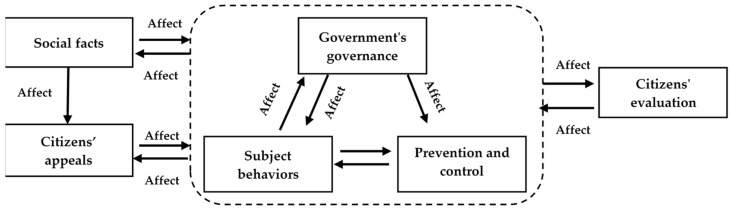
The basic model of online public opinion on the COVID-19 epidemic.

Social facts are relatively independent, a description of social reality and the source of online public opinion during the epidemic. They will directly affect various other areas, and will also give feedback to online public opinion in some aspects. Citizens’ evaluation reflects the government’s response to citizens’ appeals to a certain extent. In some cases, citizens have no special demands and will also evaluate epidemic prevention and control. Citizens’ evaluation is a reference evaluation standard generated under specific prevention and control measures and actions of various subjects. These evaluation results will in turn affect the implementation of national prevention and control measures and the actions of various subjects. Government’s governance is the action taken by the government at a relatively macro level, and it is the embodiment of the country’s risk management capability and governance attitude. Prevention and control measures reflect government governance to a certain extent, and there is a close relationship between the two. To sum up, this study draws the basic model of online public opinion on the COVID-19 epidemic as shown in Figure 3.

According to the theoretical coding results, we find that the online public opinion mechanism is based on social facts, citizens’ appeals as the starting point, subject behaviors and prevention and control measures as the focus, government’s governance as macro-control and citizens’ evaluation as the guide.

The examples of coding process using grounded theory is shown in Table 5.

### 4.4. Theoretical Saturation Test

Through three-level coding, this study obtained a theoretical model of online public opinion from the outbreak to the dissipation period of the event. In order to test the reliability of the theoretical model, the reserved network text in April 2022 (48 pieces of information) was removed from the meaningless information and then re-encoded with three-level coding. The resulting concepts were included in six categories, which was consistent with the theoretical model. The overall test results show that the theoretical model of the public opinion system is saturated.

### 4.5. Results Illustration

Based on the above research results, especially the basic model of online public opinion, it was found that the operation mechanism of the online public opinion system under the COVID-19 epidemic was mainly affected by the interaction of online public opinion objects, online public opinion subjects, online public opinion intermediaries and government forces.

The objects of online public opinion are the stimulus that triggers public opinion, which directly leads to the occurrence and spread of online public opinion. During the outbreak of COVID-19, some events related to the epidemic are likely to become “social facts” that trigger public opinion. The online public opinion objects can attract the attention of the public opinion subjects in the evolution of public opinion, and have close interests with the subjects. COVID-19 has posed a major threat to the lives and health of the masses, is a major challenge to economic and social development, and has formed “social facts” that netizens are most concerned about. As the online public opinion has a certain influence, it will have an impact on the online public opinion objects, and may also derive a series of secondary public opinion events. For example, the public is concerned about the low vaccination rate of the elderly (over 60 years old). In order to actively carry out vaccination for the elderly, the government has accelerated the pace of vaccination. However, whether some elderly people with basic diseases should be vaccinated has become a new public opinion focus. Primary public opinion events and secondary public opinion events together constitute the objects of online public opinion.

The subjects of online public opinion play a leading role in practice: especially, the evaluation results of netizens will affect the development of events. Online public opinion subjects include not only netizens, but also ordinary citizens, medical workers, government officials, etc. related to public opinion events. Netizens are the most important part of internet public opinion subjects. The netizens’ concerns, attitudes and emotions will play a leading role in the evolution of the entire public opinion system. As a non-negligible part of the public opinion subject, opinion leaders have played an increasingly important role. Some opinion leaders’ misconduct has directly played a negative role in guiding the development of the whole public opinion event. In the study, it was found that the subject of online public opinion is an unstable but instructive part among all parties involved in online public opinion.

An online public opinion intermediary is the carrier on which online public opinion comes into being and changes. The online public opinion intermediary mainly refers to information technology and major network information platforms. In this study, we found that most of the online public opinion events of COVID-19 rely on the spread of public information platforms such as microblogs, WeChat, Tik Tok and the online community, which together constitute the backbone in the dynamic evolution of online public opinion. The online public opinion intermediary is not only a platform for netizens to express their opinions through We Media, but also a platform for official mainstream media to release news and express their attitudes. It is crucial to make good use of online public opinion intermediaries for the trend of online public opinion.

Government power plays an important role in the evolution of online public opinion. From holding a government press conference to taking administrative measures, every move of the government affects the fluctuation of online public opinion events. In the context of the COVID-19, netizens are looking forward to seeing a government that is active and responsive. In the theoretical model, the arrangement of government prevention and control measures will directly affect the development of original public opinion events, and the government’s direct or indirect publicity and education will have a direct impact on the values and behaviors of online public opinion subjects. For example, the government urgently allocates living materials to ensure the basic life of residents. These actions have received positive comments from netizens and can create a good atmosphere of online public opinion.

## 5. Discussion

The development and change of online public opinion is a complex process, and online social media has greatly released the people’s right to speak. Anyone can express their demands through Weibo, WeChat, online communities and other platforms, and the people’s demands are continuously amplified through the influence of the internet. Only by recognizing, grasping and making good use of online public opinion and finding the mainstream public opinion, can the government interact with the public in a targeted manner. This will also help the government to control social risks and better achieve the effect of concerted efforts to fight the epidemic.

### 5.1. Identify and Properly Handle Events That May Trigger Online Public Opinion

The government should actively take measures to prevent negative online public opinion and actively prevent and resolve social risks. Through the analysis of online public opinion data in the first quarter of 2022, we find that as a “barometer” of social sentiment, online public opinion can directly reflect the “explicit demand” of society, which is generally manifested as new claims caused by interest damage and attempts to seek relief. Online public opinion is caused by social events, so the government must take countermeasures against events that may cause online public opinion.

The government should identify and prevent the upcoming events that may cause negative online public opinion. This is conducive to taking effective measures from the source to prevent and resolve potential social conflicts in a timely manner. On the one hand, before taking epidemic prevention and control measures, the government should assess the possible impact of the measures on people and take preventive measures in advance to minimize the adverse impact. On the other hand, the government should establish a public opinion monitoring mechanism, collect information through multiple channels, grasp social risks and hidden dangers and timely identify events that may lead to negative public opinion. Intervention shall be made in advance to prevent the risk from expanding.

For the events that have occurred, the government should actively pay attention to the evolution of the events and take positive actions to avoid causing more negative online public opinion. For some events that cause negative online public opinion, it is necessary to analyze the causes of the events in a timely manner, evaluate the hazards and impacts and find the parties involved. In short, the government should quickly meet the expectations of the masses and actively respond to their concerns.

### 5.2. Improve the Accuracy of Public Opinion Identification

Although online public opinion is not equal to public opinion, it is necessary to identify mainstream public opinion by identifying online public opinion. Accurately identifying online public opinion is of great significance, and is the key to improving the government’s risk management capabilities. In the above, we have explored and analyzed the model of online public opinion, and we can approach the mainstream public opinion by analyzing “citizens’ appeals“ and “citizen evaluation”.

First, identify vague online public opinion. Online public opinion is often ambiguous. For example, for some epidemic prevention and control measures, netizens expressed their complaints and dissatisfaction through the network platform, but did not further clarify their demands [40]. This work requires analyzing what netizens mean through the network platform in combination with the situation at that time. Only clear opinions can provide specific guidance for government decision-making.

Second, identify impulsive online public opinion. The conflict of online public opinion refers to differences in opinions among different groups, holding different views and positions on the same event. The netizen group itself is complex and diverse. Different netizens have different information, and everyone’s interests are also different. When expressing opinions on an event, different netizens have different opinions. For example, some people think that dancing for nucleic acid testing staff to express their gratitude is very warm, but some people—especially those who are carrying out nucleic acid testing—will think that doing so will affect the normal order of nucleic acid testing [41]. This requires judging which people’s demands or views are reasonable and legitimate, responding to reasonable and legitimate demands and guiding unreasonable demands.

Third, accurate identification of public opinion also needs to be combined with the basic theoretical model of online public opinion of COVID-19. Before judging the public opinion on the internet, we need to first analyze the social facts, mainly focusing on the development and changes of the epidemic, such as the scope of the spread of the epidemic, the new number of infected people, the status of social order (inflation, material shortage), and so on. This is the premise of obtaining real public opinion. When we have a basic grasp of social facts, we should pay attention to the demands of citizens, which are also the needs of citizens in the process of the development and change of the epidemic. The needs here are more manifested in the fact that they cannot be achieved by relying on their own strength. For example, due to the rapid development of the epidemic, people began to panic, so some rushed to supermarkets to buy supplies, resulting in a temporary shortage of supplies. Many citizens urgently need to buy vegetables, rice, flour and other necessities of life. When confirming the needs of citizens, we must also eliminate some unreasonable needs. For example, some netizens proposed on the internet that they need to purchase materials with reserves exceeding their actual needs. This unreasonable demand is a false demand and cannot be used as a reference factor.

### 5.3. Reasonably Respond to Citizens’ Demands

How to respond to the reasonable demands of citizens is the key to the problem. On the one hand, citizens will meet their various demands during the epidemic through their own behavior, on the other hand, they also hope that the government will provide corresponding support and protection. In the case of no epidemic or no serious epidemic, the operation of the government is stable, and the conventional prevention and control measures taken will not cause major social unrest. However, when the epidemic changes, people’s demands will be stronger and their behaviors will be more intense. Meanwhile, if the government still adopts the usual practices, it will easily lead to social instability. We found that some public opinions are generated in the process of the government’s nucleic acid testing of all employees, and there will be new demands of citizens here, such as the need for home testing of the disabled and the desire to provide sunshades for those who test outdoors. These problems will not occur when citizens voluntarily go to the testing institutions when they have their own needs. In these abnormal circumstances, the government must take emergency measures, preferably in advance. According to important information such as social facts and citizens’ demands, the government needs to formulate and improve epidemic prevention and control policies at the macro level, and take appropriate measures in the implementation of specific policies. The effectiveness of the measures taken by the government is directly reflected in the evaluation of citizens. Therefore, in the process of taking measures, we need to pay attention to the evaluation of citizens in time and make flexible adjustments to policies.

### 5.4. Keep Good Communication with Netizens

The government should make good use of online public opinion intermediaries, keep information open and transparent, and maintain information communication with internet users. During the outbreak of COVID-19, people were eager to obtain some important information. The information released by the media with a government background has the characteristics of strong authority, high reliability and great influence. It can play a positive role in guiding the online public opinion by being a timely voice throughout the epidemic. If the government fails to release the information in time, it is likely to cause panic or misunderstanding among the people. There is also the possibility that online public opinion may be misled. Opinion leaders on the internet have an important impact on the development of online public opinion. Their anomie words and deeds will directly play a negative guiding role in the development of the whole public opinion event [42]. For example, they may broadcast some unverified or non-existent events in the form of news, misleading people and bringing social risks. In order to prevent this risk, the government needs to monitor the dynamics of the network platform and accurately identify the online public opinion that has attracted wide attention, especially the public opinion caused by rumors, in a timely manner. As for online rumors, the government needs to clarify the truth in a targeted way and respond to the public’s concerns about the development of the situation in a timely manner.

### 5.5. Limitations

This brief report does not cover the government’s management of online information. The amount of public opinion in the study may not represent the actual number of public opinion events in the first quarter, which may be because some negative online public opinion has been effectively controlled and can no longer be queried.

## 6. Conclusions

The scientific analysis of online public opinion is an important tool to identify and manage risks and improve the quality of government activities. From the perspective of the modernization of national governance, online public opinion should not be regarded as a threat to risk management, but as a valuable information resource. The establishment of bottom-up information feedback channels is an important mechanism to feedback risks in the social governance system [43]. Online public opinion has the function of assisting government decision-making, and the government can identify the important information reflected in it, especially the mainstream public opinion, as a reference for decision-making. The research obtained in the online public opinion structure during COVID-19 and the online public opinion is the result of the mutual use of the object, subject, intermediary and government power of the online public opinion. It is based on social facts and citizens’ appeals as the starting point, subject behaviors and prevention and control measures as the focus, government’s governance as macro-control and citizens’ evaluation as the guide. The government should identify and properly handle events that may trigger online public opinion, and find the mainstream public opinion through identification. The government should also be rational in dealing with the mainstream public opinion and respond reasonably to the reasonable needs of people. It needs to balance the interests of different subjects, respond to the reasonable demands of citizens in a timely manner, properly handle the unreasonable demands of citizens and strive to maximize the social and public interests [44]. The government needs to fully consider the impact of various measures on public opinion, take appropriate measures in a timely manner, improve its governance abilities during the COVID-19 and especially pay attention to the interest network media and maintain communication with people in order to avoid new negative public opinion.

Through the coding analysis of online public opinion samples, we obtained a basic model of online public opinion. This model is based on summarizing node information from many online public opinion events and abstracting the ontology structure of online public opinion. This study makes it possible to analyze and manage online public opinion, and provides a new tool for online public opinion research. The model is simple and easy to understand. On this basis, we can further explore the analysis tools of complex online public opinion. Combined with the logical relationship among the elements, we propose a method to analyze online public opinion by using this model, which provides suggestions for the government for properly handling online public opinion, and provides a new scheme for the public sector to optimize epidemic prevention policies and take measures.

## Figures and Tables

**Table 1:** List of data sources.

Date	Wechat	Online Communities	Sina Weibo	Search Engine	Total
0101–0106	3	1	2	3	9
0107–0113	2	3	1	3	9
0114–0120	1	3	1	2	7
0121–0127	1	2	2	1	6
0204–0210	0	0	0	2	2
0211–0217	1	0	1	2	4
0218–0224	1	0	1	0	2
0225–0303	0	0	0	0	0
0304–0310	1	0	1	1	3
0311–0317	5	1	2	5	13
0318–0324	1	2	0	5	8
0325–0331	5	2	1	3	11

**Table 2:** Online public opinion information related to the COVID-19 epidemic on four online platforms (25–31 March 2022).

Platform	Ranking	Online Public Opinions	The Level of Public Attention
Wechat	1	● Children dance for epidemic prevention workers to thank.	4669
	2	● Changchun apologizes to the public for the difficulty of buying vegetables.	4123
	4	● Shanghai Mengshan Vegetable Market responded that 2 cabbages were sold for 93 yuan.	3440
	5	● A man in Fuxin, Liaoning Province, was sentenced to 9 months in prison for hurting workers after being prevented from jumping in line for nucleic acid testing.	3006
	6	● It is reported on the Internet that an elderly man in Shanghai was unable to go out to dispense medicine due to isolation. Family members: Please do not spread rumors.	2875
Online Communities	2	● Cap the cost to make COVID-19 antigen testing more affordable.	713
	7	● Thanks to the medical staff, don’t sing and dance at the epidemic prevention site.	656
Sina Weibo	6	● A nurse in Shanghai died of asthma: the emergency department of Shanghai Oriental Hospital was temporarily closed due to epidemic disease.	436,151,223
Search Engine	1	● Do not come to Shanghai or leave Shanghai unless necessary.	776,339
	4	● Changchun apologized to the public for the difficulty of buying vegetables, and the government has organized multiple channels to increase stocking efforts.	383,480
	7	● Langfang CDC apologizes for Anci District’s request to cull indoor animals of COVID-19 patients, which has been stopped.	311,115

**Table 3:** Examples of open coding.

Concepts	Quantity	Cases
Behaviors of epidemic prevention personnel	66	● Epidemic prevention staff shall collect personal information of epidemic related personnel within the scope permitted by law.
Epidemic prevention and control policies	61	● Negative nucleic acid test certificate within 48 h is required to enter or return to Beijing.
Changes in the epidemic situation	51	● From 0–24:00 on January 12, 76 new local confirmed cases were reported in Henan Province.
Negative evaluation	47	● Many citizens reported that “Xi’an Yimatong has collapsed again” and nucleic acid testing could not be carried out.
Take control measures for specific areas or people	45	● In order to prevent the spread of the epidemic, Tianjin immediately controlled Xinzhuang Town, where the epidemic occurred.
Strengthen support for epidemic prevention and control	42	● The government has prepared sufficient testing materials.
Provide life service guarantee	31	● Distribute emergency food such as instant food, ham sausage, mustard greens to quarantined persons.
Positive evaluation	24	● Thanks to the central government for building isolation facilities and makeshift hospitals for Hong Kong.
Report the situation to the relevant departments	23	● Some residents reported that during the epidemic, merchants who entered the community to sell vegetables were suspected of being short of food.
Release information to the public	23	● On January 17, Shenzhen held a press conference to update the first discovery of “Omicron”.

**Table 4:** Examples of axle coding.

Social Facts	Citizens’ Appeals	Subject Behaviors	Prevention and Control Measures	Citizens’ Evaluation	Government’s Governance
Changes in the epidemic situation	Nucleic acid testing requirements	Official’s enforcement actions	Investigating epidemic information	Negative evaluation	Clarifying policy principles and directions
Social order status	Medical services demands	Official’s aiding actions	Publishing epidemic alerts	Positive evaluation	Formulating prevention and control policies
Citizen health status	Information demands	Media actions	Providing life service guarantee		Releasing epidemic information
	Report the situation to the relevant departments	Citizens’ rights protection actions	Nucleic acid testing for all staff		Stabilizing market order
	Life security needs	Citizens’ illegal acts	Taking prevention and control measures for specific areas		Strengthening the support of epidemic prevention and control
	Security demands	Behaviors of epidemic prevention personnel	Taking quarantine measures for specific people		

**Table 5:** The examples of coding process.

No.	Typical Concepts	Open Coding	Axle Coding	Theoretical Coding
1	Increase in infections	Changes in the epidemic situation	Social facts	The process of forming theory
2	Traffic was restricted	Social order status		
3	Have symptoms of illness	Citizen health status		
4	Long waiting time for nucleic acid testing	Nucleic acid testing requirements	Citizens’ appeals	
5	Hospital suspended outpatient service	Medical service demands		
6	Would like to know the opening time	Information demands		
7	Called the regional office	Report the situation to the relevant departments		
8	Lack of food and water	Life security needs		
9	Unlawful restriction of personal freedom	Security demands		
10	Take measures of detention	Official’s enforcement actions	Subject behaviors	
11	Providing living materials for free	Official’s aiding actions		
12	Interview patients	Media actions		
13	Appeal to relevant departments	Citizens’ rights protection actions		
14	Violation of epidemic prevention regulations	Citizens’ illegal acts		
15	Assist in patient transfer	Behaviors of epidemic prevention personnel		
16	Investigation on epidemic prevention and control	Investigating epidemic information	Prevention and control measures	
17	Send SMS to remind citizens to improve their awareness of protection	Publishing epidemic alerts		
18	Provide emergency food	Providing life service guarantee		
19	Regional nucleic acid testing	Nucleic acid testing for all staff		
20	Take measures to close the building	Taking prevention and control measures for specific areas		
21	Take isolation measures for cases	Taking quarantine measures for specific people		
22	Anger at the government’s actions	Negative evaluation	Citizens’ evaluation	
23	Thank the medical staff	Positive evaluation		
24	Propose the dynamic zero COVID-19 strategy	Clarifying policy principles and directions	Government’s governance	
25	Release the latest policy of entering and returning to Beijing	Formulating prevention and control policies		
26	Hold a press conference	Releasing epidemic information		
27	Ensuring the supply of living materials	Stabilizing market order		
28	Provide convenient nucleic acid testing services	Strengthening the support of epidemic prevention and control		

## Data Availability

The data used and analyzed during the current study are available from the corresponding author upon reasonable request.
